# Overexpression of miR-489 enhances efficacy of 5-fluorouracil-based treatment in breast cancer stem cells by targeting XIAP

**DOI:** 10.18632/oncotarget.22985

**Published:** 2017-12-06

**Authors:** Xuedong Wang, Xinguo Wang, Juan Gu, Ming Zhou, Zhimin He, Xinhui Wang, Soldano Ferrone

**Affiliations:** ^1^ Department of Medical Laboratory Science, The Fifth People’s Hospital of Wuxi, The Medical School of Jiangnan University, Wuxi, Jiangsu 214005, China; ^2^ Department of Pathology, The Fifth People’s Hospital of Wuxi, Nanjing Medical University, Wuxi, Jiangsu 214005, China; ^3^ Cancer Research Institute, Central South University, Changsha, Hunan 410078, China; ^4^ Cancer Hospital and Cancer Research Institute, Guangzhou Medical University, Guangzhou, Guangdong 510095, China; ^5^ Department of Medical Oncology, Massachusetts General Hospital, Harvard Medical School, Boston, MA 02114, USA

**Keywords:** BCSCs, miR-489, 5-fluorouracil, resistance, XIAP

## Abstract

Population of cancer stem cells (CSCs) in breast cancer is reported to be resistant to chemotherapy. Furthermore, many cases of treatment failure are induced by the chemoresistance of CSCs in breast cancer patients. Therefore, novel strategies should be explored urgently to reverse drug-resistance in breast cancer stem cells (BCSCs). In this study, we isolated and cultured the BCSCs from the T-47D and SKBR3 breast cancer cell lines. We observed significant resistance to 5-fluorouracil in BCSCs. Mechanically, we found that expression of miR-489 was decreased in BCSCs. Furthermore, overexpression of miR-489 was found to increase the cytotoxicity of 5-fluorouracil to BCSCs. XIAP, a key anti-apoptotic protein, was proved to be the target of miR-489. We found that enforced expression of XIAP through its recombinant expression vector abolished the effect of miR-489 on reversing the 5-fluorouracil resistance. On the contrary, embelin, a XIAP specific inhibitor, was found to sensitize BCSCs to 5-fluorouracil similarly with miR-489. In summary, our data demonstrate that introduction with miR-489 represents a novel strategy to enhance efficacy of 5-fluorouracil-based treatment in BCSCs.

## INTRODUCTION

Breast cancer represents a common malignant tumor, which usually leads to a high mortality rate in women worldwide [1]. Recently, CD44^+^/CD24^-/low^ phenotype of breast cancer cells are reported to exhibit highly tumorigenic and self-renewing capability. This population of tumor cells are called breast cancer stem cells (BCSCs) [2, 3]. BCSCs initiate tumors and drive tumor growth and metastasis. Moreover, BCSCs are found to be resistant to chemotherapy. Many cases of treatment failure are induced by the chemoresistance of CSCs in breast cancer patients [4, 5, 6, 7]. Thus, it’s urgent to develop CSC-targeting therapies to enhances efficacy of chemotherapy in breast cancer.

5-Fluorouracil (5-FU) is one kind of efficient anti-cancer drugs for treatment of various tumors including breast cancer in standard chemotherapy protocols. Cellular 5-FU can be transformed into 5-oligodeoxynucleotide, which inhibits the activity of thymidine synthase and thus inhibiting DNA synthesis. Therefore, 5-FU is known to induce cell apoptosis in tumors including breast cancer [8, 9, 10]. However, chemoresistance against 5-FU induced by long-term use of 5-FU has become a major concern [11]. It’s urgent to explore novel chemotherapeutic adjuvants which are able to decrease the 5-FU-resistance.

MicroRNAs (miRNAs) are a new class of non-coding RNAs that modulates the expression of targeting genes through binding to the 3’-untranslated region (3’-UTR) of mRNA [12, 13]. Studies demonstrate that about 60% of the human genes can be potentially regulated by miRNAs. Therefore, they participate in various cellular processes such as cell proliferation, differentiation, and apoptosis. Moreover, dysregulation of miRNAs also has been reported to be involved in tumorigenesis and chemoresistance in tumors including breast cancer [14, 15, 16, 17]. Thus, correcting the disorder of miRNAs may represent a potential strategy to enhance the efficacy of chemotherapy in breast cancer. Increasing researches demonstrate that CSCs are resistant to 5-FU treatment, and great efforts have been made to decrease the chemoresistance against 5-FU [18, 19]. In this study, novel strategy of introduction with miR-489 was tested to try to reverse the 5-FU-resistance in BCSCs.

## RESULTS

### BCSCs showed resistance to 5-FU treatment

**Figure 1:**
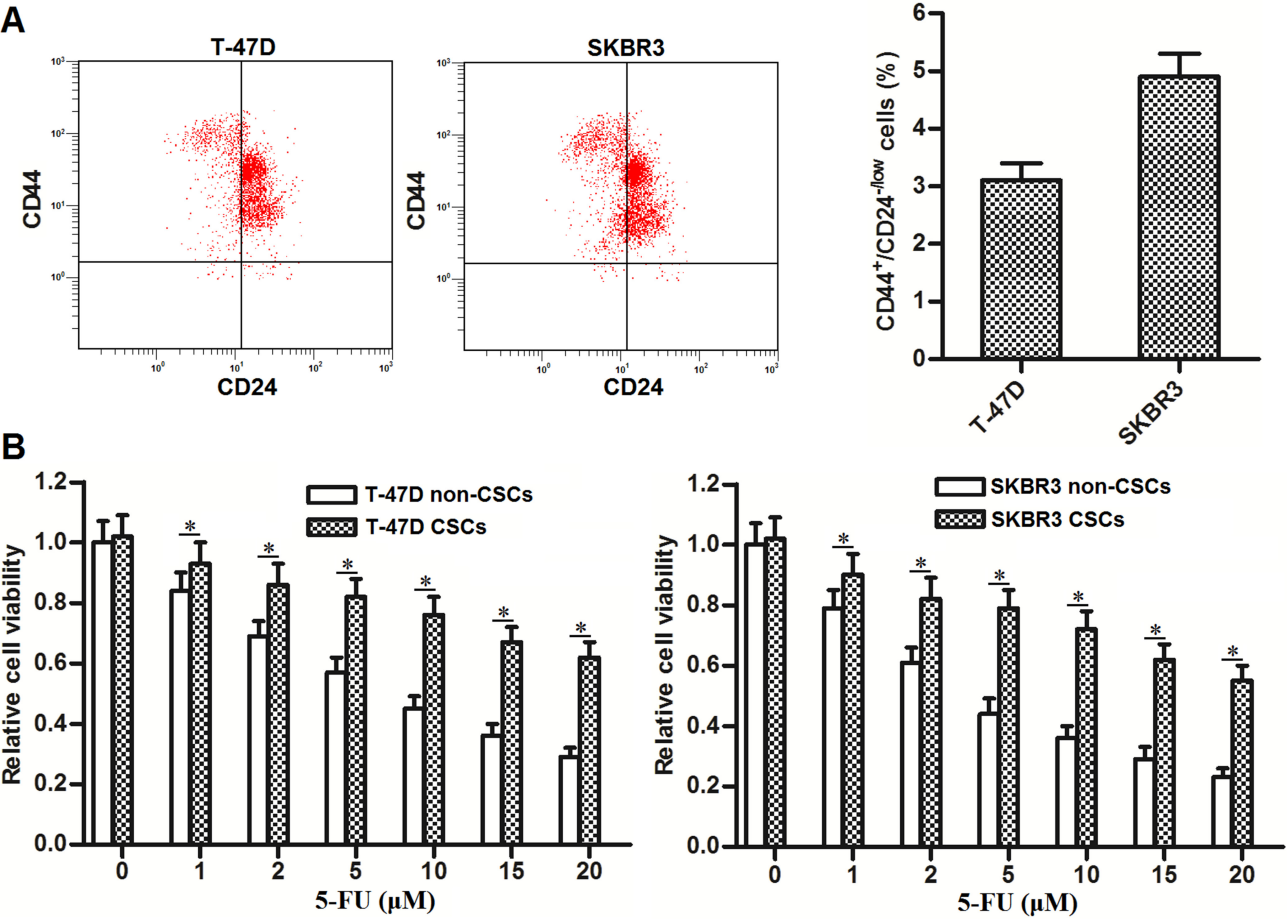
BCSCs show resistance to 5-FU treatment (**A**) Flow cytometry was performed to analyze the CD44^+^/CD24^-/low^ phenotype of BCSCs in T-47D and SKBR3 cell lines. (**B**) T-47D and SKBR3 CSCs and non-CSCs were treated with different concentrations of 5-FU (0∼20 μM). Forty-eight hours later, MTT assays were performed to evaluate the sensitivity of T-47D and SKBR3 CSCs and non-CSCs to 5-FU treatment. ^*^P < 0.05.

To study the sensitivity of BCSCs to 5-FU treatment, we firstly separated the cancer stem cell population of CD44^+^/CD24^-/low^ phenotype [2, 3] in T-47D and SKBR3 cell lines. As shown in Figure 1A, the proportions of CSCs in these breast cancer cell lines are approximately three to five percent. After separating and culturing these BCSCs, we next performed MTT assays to investigate the resistance of T-47D and SKBR3 CSCs to 5-FU treatment. As shown in Figure 1B, we found that T-47D and SKBR3 CSCs were tolerant to 5-FU treatment compared to T-47D and SKBR3 non-CSCs, respectively. These results demonstrated the resistance of BCSCs to 5-FU treatment.

### Expression of miR-489 was decreased in BCSCs

**Figure 2:**
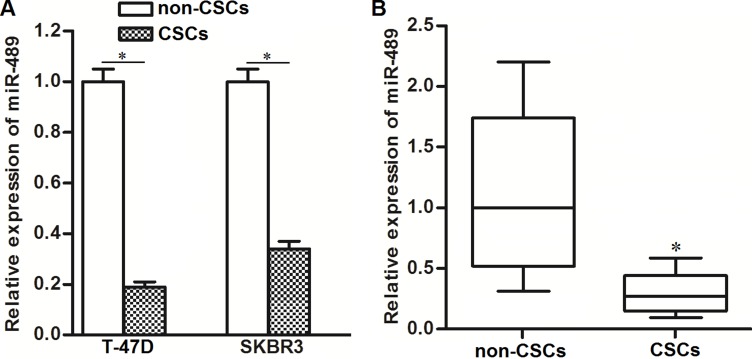
Expression of miR-489 was decreased in BCSCs (**A**) QRT-PCR assays were performed to detect the relative expression of miR-489 in T-47D and SKBR3 CSCs and non-CSCs. ^*^P < 0.05. (**B**) Breast cancer patients’ tissues (*n* = 30) were digested by collagenase type III. CSCs and non-CSCs in cell suspension were separated flow cytometry. Subsequently, relative expression of miR-489 in these CSCs and non-CSCs was detected by qRT-PCR analysis. ^*^P < 0.05.

Previous studies suggested that miR-489 was a potential tumor suppressor in cancer cells [20]. In our study, we observed dysregulation of miR-489 in BCSCs. As shown in Figure 2A, T-47D and SKBR3 CSCs showed significantly lower levels of miR-489 compared to T-47D and SKBR3 non-CSCs, respectively. In breast cancer patients’ samples, we also observed significantly decrease of miR-489 expression in CSCs population (Figure 2B). We therefore needed to study the potential role of miR-489 in BCSCs.

### Overexpression of miR-489 weakened the resistance of BCSCs to 5-FU treatment

**Figure 3:**
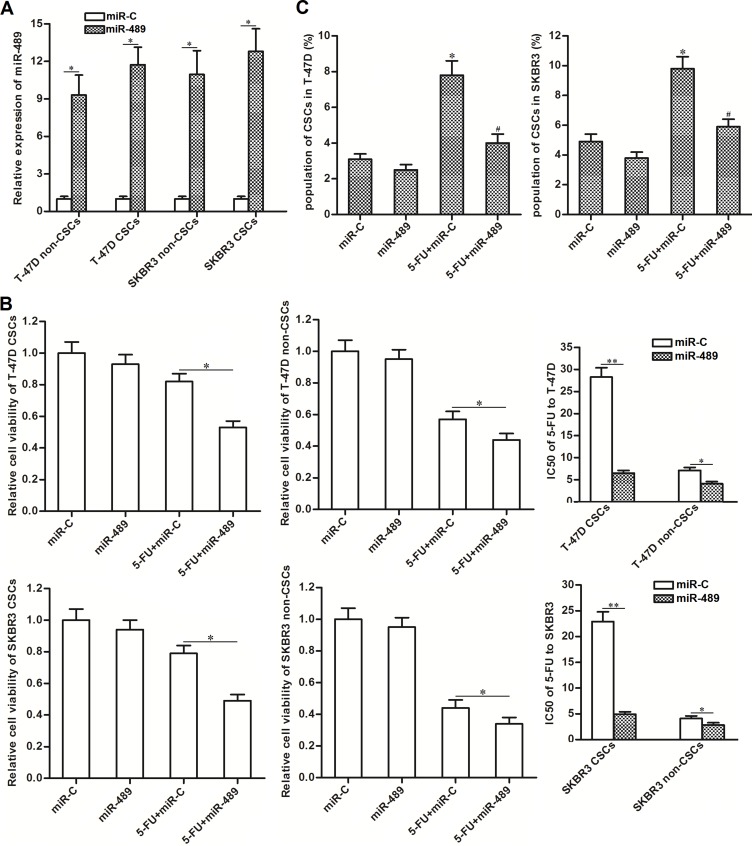
Overexpression of miR-489 decreased the resistance of BCSCs to 5-FU treatment (**A**) Transfection with miR-489 mimics increased the expression of miR-489 in T-47D and SKBR3 CSCs and non-CSCs. ^*^P < 0.05. (**B**) Overexpression of miR-489 enhanced the cytotoxicity of 5-FU (5 μM) to T-47D and SKBR3 CSCs and non-CSCs. ^*^P<0.05, ^**^P < 0.01. (**C**) Overexpression of miR-489 suppressed the enrichment of CSC population induced by 5-FU (5 μM) in T-47D and SKBR3 cell lines. ^*^P < 0.05 *vs.* miR-C group, ^#^*P* < 0.05 *vs.* 5-FU + miR-C group.

To correct the downregulation of miR-489 in BCSCs, we transfected them with miR-489 mimics. As shown in Figure 3A, transduction with miR-489 mimics significantly increased the expression of miR-489 in T-47D and SKBR3 CSCs as well as their non-CSCs. Interestingly, we observed that overexpression of miR-489 significantly enhanced the cytotoxity of 5-FU to T-47D and SKBR3 CSCs. Specifically, compared to miR-C group, transfection with miR-489 decreased the IC50 of 5-FU by 77.5 percent to T-47D CSCs and 74.8 percent to SKBR3 CSCs. On the other hand, effect of miR-489 on reducing the IC50 of 5-FU to T-47D and SKBR3 non-CSCs was obviously slighter than the T-47D and SKBR3 CSCs (Figure 3B). In addition, we found that 5-FU single treatment increased the proportion of CSC population, whereas overexpression of miR-489 significantly suppressed the enrichment of CSC population induced by 5-FU (Figure 3C). Token together, these results indicated that CSCs were more sensitive to miR-489 rather than the non-CSCs. Overexpression of miR-489 was able to enhance the cytotoxicity of 5-FU to BCSCs.

### Overexpression of miR-489 enhanced the anti-tumor effect of 5-FU *in vivo*

**Figure 4:**
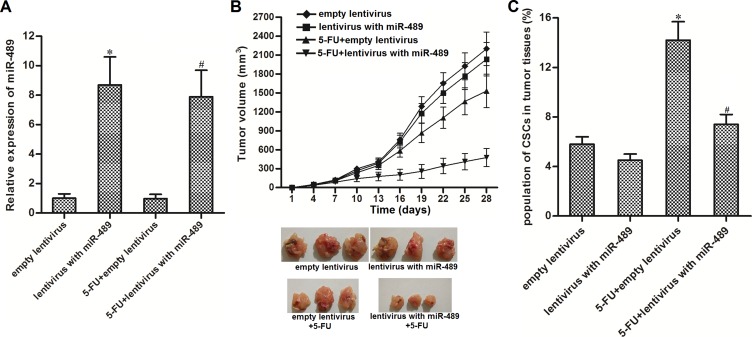
Overexpression of miR-489 enhanced the anti-tumor effect of 5-FU *in vivo* (**A**) T-47D cells were infected with lentivirus with miR-489 and subcutaneously injected into BALB/c nude mice (8 mice for each group). Expression of miR-489 in lentivirus-infected tumors was detected by using qRT-PCR analysis. ^*^P < 0.05 *vs.* empty lentivirus group, ^#^*P* < 0.05 *vs.* 5-FU + empty lentivirus group. (**B**) Tumor growth in nude mice was inhibited by co-treatment with miR-489 and 5-FU (5 mg/kg 5-FU every three days) . (**C**) 5-FU-induced enrichment of CSCs was suppressed by miR-489 overexpression in tumors. ^*^P < 0.05 *vs.* empty lentivirus group, ^#^*P* < 0.05 *vs.* 5-FU + empty lentivirus group.

We next investigated the effect of miR-489 on enhancing the anti-tumor effect of 5-FU *in vivo*. We observed that miR-489 expression was significantly higher in the groups transduced with lentivirus-miR-489 than groups transduced with empty lentivirus (Figure 4A). Furthermore, tumors overexpressed miR-489 were more sensitive to 5-FU treatment than the control tumors (Figure 4B). Interestingly, we found that 5-FU single treatment increased the proportion of CSC population in control tumors, whereas the 5-FU-induced enrichment of CSCs was obvious slighter in miR-489-overexpressed tumor (Figure 4C). Together, we demonstrated that miR-489 enhanced the anti-tumor effect of 5-FU by targeting CSCs *in vivo*.

### XIAP was the target of miR-489 in BCSCs

**Figure 5:**
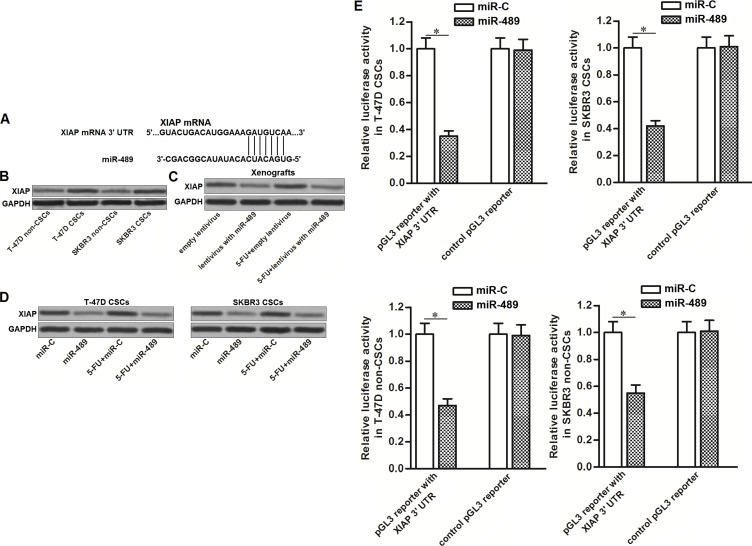
MiR-489 targeted XIAP in BCSCs (**A**) Putative binding site of miR-489 in the XIAP 3’-UTR predicted by online bioinformatics database of TargetScan. (**B**) Upregulation of XIAP in T-47D and SKBR3 CSCs compared to their non-CSCs. (**C**) Overexpression of miR-489 in breast cancer cells decreased the expression of XIAP *in vivo*. (**D**) Overexpression of miR-489 in breast cancer cells decreased the expression of XIAP *in vitro*. (**E**) Transfection with miR-489 decreased the luciferase activities of the pGL3 reporter with XIAP 3’-UTR. ^*^P < 0.05.

To investigate the mechanism by which miR-489 weakened the resistance of BCSCs to 5-FU, we searched for potential targets of miR-489 through online bioinformatics database of TargetScan, miRanda and PicTar. Among these potential targets, XIAP, an apoptosis suppressor [21], was predicted to be a target of miR-489 because its mRNA 3’-UTR contained complementary sequences paired to miR-489 (Figure 5A). Besides, the results of western blot analysis showed that expression level of XIAP was upregulated in BCSCs compared to their corresponding non-CSCs (Figure 5B). It suggested the negative correlation between miR-489 and XIAP in breast cancer. Next, we found that overexpression of miR-489 decreased the expression of XIAP in breast cancer cells, no matter whether they were treated with 5-FU *in vitro* and *in vivo* (Figure 5C and 5D). To confirm the effect of miR-489 on suppressing XIAP expression, luciferase reporter assays were performed in T-47D and SKBR3 CSCs and non-CSCs. We found that overexpression of miR-489 decreased the luciferase activities of the pGL3 reporter with XIAP 3’-UTR but not the empty one (Figure 5E). These data indicated that XIAP was the target of miR-489 in BCSCs.

### Overexpression of miR-489 decreased the resistance of BCSCs to 5-FU by targeting XIAP

**Figure 6:**
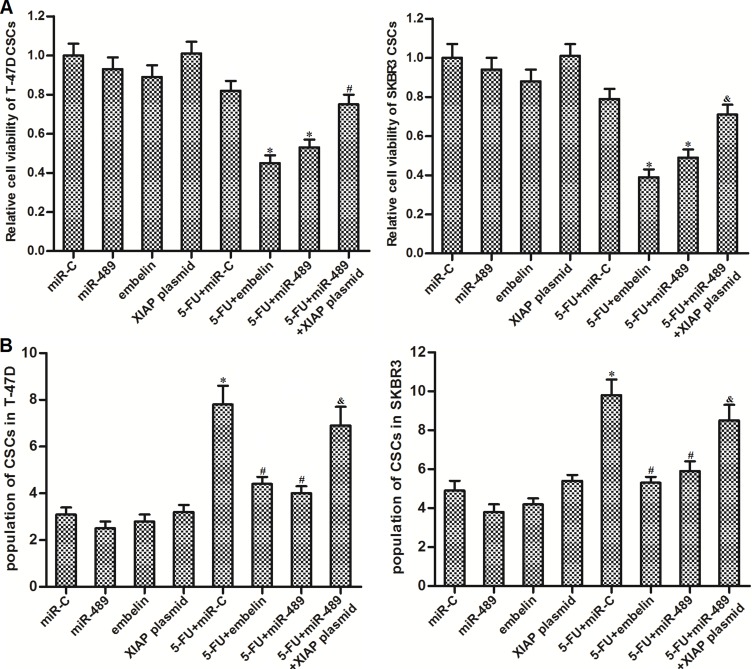
MiR-489 decreased the expression of XIAP to sensitize BCSCs to 5-FU treatment (**A**) After transfection with miR-489, miR-C and XIAP plasmid, T-47D and SKBR3 CSCs were treated with 5-FU (5 μM) and embelin (10 μM) followed by detecting the cell viability through MTT assays. ^*^P < 0.05 *vs.* 5-FU + miR-C group, ^#^*P*<0.05 *vs.* 5-FU + miR-489 group. (**B**) Unsorted T-47D and SKBR3 cells were transfected with miR-489, miR-C and XIAP plasmid followed by treating with 5-FU (5 μM) and embelin (10 μM). Population of CSCs was detected by using flow cytometry. ^*^P < 0.05 *vs.* miR-C group, ^#^*P*<0.05 *vs.* 5-FU + miR-C group, ^&^*P* < 0.05 *vs.* 5-FU + miR-489 group.

To investigate whether the effect of miR-489 on reversing the 5-FU resistance in BCSCs was mediated by XIAP repression, we restored XIAP expression in miR-489-overexpressed BCSCs. On the other hand, we used embelin which is a specific inhibitor against XIAP [22] to study the role of XIAP in 5-FU-based treatment in BCSCs. We found that XIAP restoration was able to rescue the BCSCs from the co-treatment with miR-489 and 5-FU. Besides, similarly as miR-489, embelin treatment also increased the sensitivity of BCSCs to 5-FU-based chemotherapy (Figure 6A). Additionally, similarly as miR-489, embelin repressed the enrichment of CSC population induced by 5-FU. On the contrary, XIAP plasmid abolished the effect of miR-489 on suppressing the 5-FU-induced enrichment of BCSCs (Figure 6B). These results indicated that miR-489 decreased the resistance of BCSCs to 5-FU by targeting XIAP.

### MiR-489 promoted 5-FU-induced apoptosis in BCSCs through suppressing the function of XIAP

**Figure 7:**
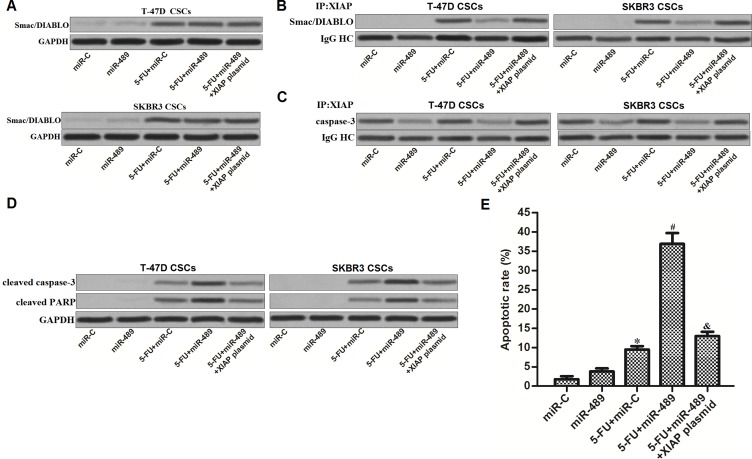
MiR-489 suppressed the function of XIAP to promote 5-FU-induced apoptosis in BCSCs (**A**) Mitochondria in T-47D and SKBR3 CSCs were removed before detection of Smac/DIABLO by using western blot assays. (**B**) Immunoprecipitation and western blot assays were performed to determine the interaction with XIAP and Smac/DIABLO. (**C**) Immunoprecipitation and western blot assays were performed to determine the interaction with XIAP and caspase-3. (**D**) After transfection with miR-489, miR-C and XIAP plasmid, T-47D and SKBR3 CSCs were treated with 5-FU (5 μM) followed by detecting the cleavage of caspase-3 and PARP by using western blot analysis. (**E**) After transfection with miR-489, miR-C and XIAP plasmid, T-47D and SKBR3 CSCs were treated with 5-FU (5 μM) followed by measuring the apoptosis using flow cytometry. ^*^P < 0.05 *vs.* miR-C group, ^#^*P* < 0.05 *vs.* 5-FU + miR-C group, ^&^*P* < 0.05 *vs.* 5-FU + miR-489 group.

Preceding results have emphasized that decrease of XIAP expression was the mechanism by which miR-489 increased the cytotoxicity of 5-FU to BCSCs. We next investigated the function of XIAP in BCSCs co-treated with 5-FU and miR-489. Smac/DIABLO is an effective apoptotic inducer in cancer cells, which can be inactivated by interacting with XIAP [23]. We found that treatment with 5-FU induced release of Smac/DIABLO in BCSCs, no matter whether the miR-489 was transfected (Figure 7A). However, because of the decrease of XIAP expression induced by miR-489, interaction with XIAP and Smac/DIABLO was significantly inhibited in 5-FU and miR-489 co-treated BCSCs (Figure 7B). Similarly, amount of inactive caspase-3 binding to XIAP was also decreased due to the miR-489 overexpression (Figure 7C). As the results, apoptotic marker of caspase-3 and its substrate of PARP were dramatically activated (Figure 7D) before the apoptosis was finally appeared (Figure 7E) in BCSCs co-treated with miR-489 and 5-FU.

## DISCUSSION

Recently, studies demonstrate that miR-489 acts as a tumor suppressor in several cancers. In human bladder cancer, miR-489 targets mRNA of JAG1, a Notch ligand, to suppress the proliferation and invasion of tumor cells [24]; In lung cancer cells, inhibition of miR-489 upregulates the expression of N-cadherin and vimentin but decrease the level of E-cadherin to promote the epithelial mesenchymal transition (EMT) [25]. Besides these anti-tumor effects, miR-489 is also reported to increase chemosensitivity to adriamycin-resistant human breast cancer cells [26]. It’s clear that miR-489 inhibits tumorigenesis and chemoresistance in routine cancer cells. However, the effect of miR-489 on cancer stem cells (CSCs) remains unclear. CSCs, which are also called tumor initial cells, are responsible for tumor formation, recurrence and chemoresistance [27]. Here, we studied the association between miR-489 and chemosensitivity of BCSCs to 5-FU.

In the present study, we observed significant downregulation of miR-489 in CSC population of both breast cancer cell lines and patients’ specimens. Furthermore, functional assays showed that overexpression of miR-489 weakened the resistance of BCSCs to 5-FU and reduced the IC50 of it to BCSCs. *In vivo* tumorigenesis assays, we found that the miR-489-overexpressed tumors were more sensitive to 5-FU treatment. Additionally, overexpression of miR-489 was able to reduce the efficacy of 5-FU to enrich the CSC population in BCSCs. It was probably because BCSCs were more sensitive to miR-489 rather than the non-CSCs. Taken together, our work suggested that overexpression of miR-489 was able to enhance the efficacy of 5-FU-based treatment in breast cancer by targeting BCSCs.

X-linked Inhibitor of Apoptosis Protein (XIAP), a member of anti-apoptotic protein family, suppresses apoptosis of cancer cells via interaction with apoptotic inducers. Functions of caspases and Smac/DIABLO which represent activators of apoptosis are controlled by cellular XIAP [28, 29]. Researches reveal that overexpression of XIAP contribute to resistance of cancer cells to clinical chemotherapeutic drugs such as 5-FU, and thus high expression level of XIAP is responsible for poor prognosis in many cancers [30, 31, 32]. As studies demonstrate that balance of XIAP and caspases/Smac/DIABLO determine the efficacy of chemotherapy [33], decreasing the level of XIAP is considered as an effective strategy to enhance the chemotherapy-induced apoptosis in cancer cells [34].

In the present study, we demonstrated that miR-489 targeted XIAP in breast cancer. As expression of XIAP was inhibited by miR-489 in BCSCs, rescuing XIAP expression by using its plasmid abolished the effect of miR-489 on reversing the chemoresistance to 5-FU. On the contrary, co-treatment with embelin which is the specific inhibitor of XIAP also sensitized BCSCs to 5-FU. However, unlike the tumor suppressor miR-489, embelin is an exogenous herbal medicine with some potential toxicity. In addition, since XIAP inactivates caspases and Smac/DIABLO, our functional assays proved that overexpression of miR-489 reduced the interaction with XIAP and caspase-3/Smac/DIABLO, and thus expanding the apoptotic signaling induced by 5-FU treatment. Token together, our work emphasized the importance of miR-489/XIAP axis in chemosensitivity. One important mechanism by which miR-489 exerted its effect on reversing the 5-FU-resistance was dependent on the downregulation of XIAP in BCSCs.

In summary, we demonstrate here that overexpression of miR-489 enhances efficacy of 5-FU-based treatment in BCSCs by targeting XIAP. However, further efforts should be made to explore the effect of miR-489 on other types of CSCs and other chemotherapeutic agents.

## MATERIALS AND METHODS

### Breast cancer cell lines and patients’ specimens

Human breast cancer cell lines T-47D and SKBR3 were purchased from ATCC (American Type Culture Collection, Manassas,VA, USA) and cultured in DMEM medium (Invitrogen, Carlsbad, CA, USA) supplemented with 10% fetal calf serum. Cells were grown at 37 °C in a humidified chamber with 5% CO2. For use of surgical specimens derived from 30 primary breast cancer patients, we obtained informed consent from all of them. Furthermore, we obtained with the approval of the ethics committee of The Fifth People’s Hospital of Wuxi, The Medical School of Jiangnan University to use these specimens.

### Cell sorting of CD44^+^/CD24^-/low^ population in human breast cancer cell lines

Previous studies demonstrate that CD44^+^/CD24^-/low^ phenotype of breast cancer cells are considered as BCSCs [2, 3]. We therefore used CD24-FITC and CD44-PE antibodies (BD Pharmingen, USA) to separate the population of BCSCs from T-47D and SKBR3 cell lines and patients’ tumor tissues. Briefly, single cell suspension of breast cancer incubated with CD24-FITC and CD44-PE antibodies on ice. Subsequently, the incubated cells were analyzed on flow cytometry. CD44^+^CD24^−/low^ BCSCs were purified by using the fluorescent-activated cell sorting equipment (Beckman Coulter, USA).

### Quantitative real-time polymerase chain reaction (qRT-PCR) for miR-489 expression

Total RNAs in T-47D and SKBR3 cell lines and patients’ tumor tissues were isolated by using TRIzol reagent (Invitrogen). Reverse transcription reaction for miR-489 was performed by using miR-489 specific stem-loop primer. The sequence was as follows: 5’-CTCAACTGGTGTCGTGGA GTCGGCAATTCAGTTGAGGCTGCCGT-3’. After reverse transcription of miR-489, PCR reaction was performed to detect the miR-489 expression by using SYBR® Premix Ex Taq™ II (Takara, Dalian, China) according to the manufacturer’s protocols. U6 snRNA was used as the internal control to normalize the relative expression of miR-489.

### Cell transfection

MiR-489 mimics (5′-GUGACAUCACAUAUACGGCAGC-3′) and random RNA oligonucleotides (miR-C, 5′-ACUGAAUACUGCGCAAACGUGC-3′) were purchased from RiboBio Co. Ltd. (Guangzhou, China).XIAP open reading frame was amplified and then linked to the pcDNA3.1 eukaryotic expression vector (Invitrogen) to overexpress the gene of XIAP in BCSCs. For transfection,cells were cultured in 6-well plates overnight followed by transfecting with miR-489 mimics (50 pmol/ml), miR-C (50 pmol/ml) and XIAP plasmid (2 μg/ml) by using Lipofectamine 2000 (Invitrogen) according to the manufacturer’s protocols. After transfection, cells were collected for the following experiments.

### Cell viability assay

After transfection with miR-489, miR-C and XIAP plasmid, cells were seeded on 96-well plates with a concentration of 5 × 10^3^ cell/well. Subsequently, cells were treated with 5-FU for forty-eight hours. After that, 20 μl of 3-(4, 5-dimethylthiazol-2-yl)-2, 5-diphenyltetrazolium bromide, MTT, Sigma-Aldrich, USA) was added to the cells (5 mg/mL) and incubated at 37 °C for four hours. After incubation, MTT was removed, while absorbance at 570 nm was measured when cells were suspended in 150 μL of dimethyl sulfoxide (DMSO).

### Luciferase reporter assay

Fragment of human XIAP mRNA 3’-UTR was cloned into pGL3 Luciferase Reporter Vector (Promega, USA). For luciferase reporter assays, cells were co-transfected with pGL3 reporter with XIAP 3’-UTR and miR-489 mimics or miR-C. Forty-eight hours later, luciferase activities were measured by using Dual Luciferase Assay System (Promega) according to the manufacturer’s instructions.

### Mitochondria removal

To obtain the protein of Smac/DIABLO in cytoplasm, mitochondria of BCSCs were removed by using Mitochondria/Cytosol Fraction Kit (BioVision, USA) according to the manufacturer’s instructions.

### Immunoprecipitation and western blot

For immunoprecipitation, cytoplasm of BCSCs was probed with anti-XIAP (Cell Signaling Technologies, USA) overnight at 4°C. After that, protein A agarose beads were supplemented and maintained for two hours before they were collected by centrifugation. Subsequently, proteins on beads were released through boiling in sodium dodecyl sulfate (SDS) sample buffer. For western blot assay, proteins in cytoplasm of BCSCs were separated by 10% SDS-PAGE before they were transferred to nitrocellulose membranes. Subsequently, membranes were probed by anti-XIAP, anti-Smac/DIABLO, anti-caspase-3, anti-cleaved caspase-3, anti-cleaved PARP, anti-IgG HC and anti-GAPDH (Cell Signaling Technology) overnight at 4°C. Proper secondary antibodies were used to conjugate with the primary antibodies before the signals were detected by using enhanced chemilu-minescence detection kit (Pierce, USA).

### Apoptosis analysis

Cells were transfected with miR-489, miR-C and XIAP plasmid before they were treated with 5 μM 5-FU. After treatment, cells were collected and then stained with Annexin-V and Propidium iodide (PI) according to the manufacturer’s instructions (Sigma-Aldrich, USA). Cell apoptosis was analyzed by using flow cytometry.

### *In vivo* tumorigenesis assays

MiR-489 precursor sequences were amplified and linked to lentiviral vectors. Subsequently, lentivirus with miR-489 was packaged by using lentiviral packaging kit (Genechem Co. Ltd, Shanghai, China). For *in vivo* tumorigenesis assays, T-47D cells were infected with 1 × 10^6^ units of lentivirus with miR-489 (or empty lentivirus acted as control). Then, 5 × 10^6^ lentivirus-infected T-47D cells were subcutaneously injected into female BALB/c nude mice (4-5-week-old). 5-FU was given through intraperitoneal injection every three days (5 mg/kg) after xenografts reached 0.5 cm in diameter. Xenograft size was measured every 3 days and calculated based on the equation of 1/2 × length × width^2^. All mice were sacrificed on day 28 days. The animal care and experimental protocols were approved by the Animal Care Committee of The Fifth People’s Hospital of Wuxi, The Medical School of Jiangnan University.

### Statistical analysis

Data were obtained from at least three separate experiments and presented as mean ± standard deviation (SD). Statistical analysis was performed by Student’s t test for two groups comparison and one-way ANOVA for Multiple groups comparison. Comparisons were analyzed by using SPSS 15.0 software, and values of *P* < 0.05 were considered statistically significant.
